# Emergency Abdominal Surgery in the Elderly: Can We Predict Mortality?

**DOI:** 10.1007/s00268-016-3751-3

**Published:** 2016-10-25

**Authors:** Anna E. Sharrock, Jenny McLachlan, Robert Chambers, Ian S. Bailey, James Kirkby-Bott

**Affiliations:** 1 Emergency General Surgery Department, Southampton General Hospital, Southampton, UK; 2 Anaesthetics Department, Southampton General Hospital, Southampton, UK; 3 General Intensive Care Department, Southampton General Hospital, Southampton, UK; 4 Inflammation, Repair and Development Section, National Heart and Lung Institute, Imperial College, London, UK

## Abstract

**Background:**

The United Kingdom population is ageing. Half of patients requiring an emergency laparotomy are aged over 70, 20 % die within 30 days, and less than half receive good care. Frailty and delay in management are associated with poor surgical outcomes. P-POSSUM risk scoring is widely accepted, but its validity in patients aged over 70 undergoing emergency laparotomy is unclear. Aims: To assess if P-POSSUM risk stratification reliably predicts inpatient mortality in this group and establish whether those who died within 30 days received delayed care.

**Methods:**

Observational study of consecutive patients aged 70 and over fulfilling the National Emergency Laparotomy Audit criteria from a tertiary hospital. The predictive value of pre-operative P-POSSUM, ASA, lactate and other routine variables was assessed. Surgical review, decision to operate, consultant surgical review, antibiotic prescription, laparotomy and discharge or death time points were assessed by 30-day survival.

**Results:**

One hundred and ninety-three patients were included. This represented 46.28 % of those undergoing an emergency laparotomy in our centre. Pre-operative P-POSSUM scoring, ASA grade and lactate were moderate predictors of mortality (AUC 0.784 and 0.771, respectively, lactate AUC 0.705, all *p* ≤ 0.001). No correlation existed between pre-operative P-POSSUM and days to death (*p* = 0.209), nor were there delays in key management timings in those who died in 30 days.

**Conclusions:**

P-POSSUM scoring may predict inpatient mortality with moderate discrimination. Addition of frailty scoring in this high-risk group might better identify those with a high risk of mortality after emergency laparotomy and would be a fertile area for further research.

## Introduction

In the United Kingdom (UK), the median age of the population is increasing [1], and 10.15 % will be aged 75 and over by 2024 [2]. Whilst the term ‘elderly’ has not been universally defined, those aged 70 have been considered to be the lower limit for Medicine for the Care of Older People (MCOP) specialist review by the NELA Project Team [3]. Not only is the population ageing, but those over the age of 70 who undergo an emergency laparotomy have an inpatient mortality of 21.4 %, with considerable regional variation reported [4]. To put the significance of emergency abdominal surgery in the elderly into context, patients over the age of 80 who are admitted to hospital for an operation and die within 30 days will have had abdominal surgery in 31.2 % of cases and have been admitted as an emergency, rather than electively in 83.4 % of cases [5].

It has been estimated that 10–15 % of the over 80s are ‘frail’ [4], and because of the complexity of managing co-morbidities and frailty, it has been recommended that patients in general hospitals have early access to a specialist team for elderly people [6]. The definition of frailty lacks a uniting consensus [7], but descriptions include a lack of reserve or phenotype involving unintentional weight loss, exhaustion, grip-strength weakness, slow walking and low physical activity [8]. Despite the National Service Framework recommendation in 2001, the National Emergency Laparotomy Audit (NELA) has reported that fewer than 40 % of individuals over 70 were assessed by a Medicine for the Care of the Older Person (MCOP) specialist in 94 % of the 190 hospitals included in the audit [3]. This reflects the finding that care was considered to be good in only 36 % of those elderly patients who died within 30 days of surgery [5]. Good general hospital care involves early access to specialists, early MCOP review, maintenance of general health status, support of privacy and overall care quality, and appropriate training of staff [6]. Of these, clinically significant delay has been identified as one of the main contributors to less than good care [5].

Pre-operative risk assessment allows for appropriate pre-emptive resource allocation, and may aid in decision-making by, or for the patient in light of their best interests. Patients who undergo major abdominal surgery in the elective setting should be appropriately assessed to identify modifiable risk factors that can be addressed before surgery [9]. In the emergency setting, overall risk should still be assessed, to target resources, optimise physiology and direct decision-making around ongoing care, even if the opportunity to do so is limited. Several perioperative scores have been developed to aid in this process, and to facilitate audit and unit performance analysis. These include the widely used American Society of Anaesthesiology (ASA), Acute Physiology and Chronic Health Evaluation (APACHE) [10] and Physiological and Operative Severity Score for the enUmeration of Mortality and morbidity (POSSUM) [11]. The accuracy of mortality prediction in the latter was enhanced in the Portsmouth-modified (P-POSSUM) score [12], by adjusting the statistical weight placed on the physiological and operative parameters (Table 1). However, this score has been criticised, as the range of parameters included in calculating an outcome may not be routinely performed [13], and because of concerns that it overestimates mortality in those with low risk and hides poor surgical standards [14]. POSSUM and P-POSSUM scores have been directly compared, and in one cohort of 3741 surgical patients on a level 1 care ward, the area under curve (AUC) of the receiver–operator curve (ROC) was 0.81 and 0.84, respectively, indicating both had good discrimination between those who survived and those who did not [15].

**Table 1:** POSSUM parameters (modified from Prytherch et al. [12])

Physiological (severity)	Operative
Age^a^ Cardiac history Respiratory history ECG changes^a^ Systolic blood pressure Heart rate Haemoglobin White cell count^a^ Urea Sodium Potassium Glasgow Coma Scale	Complexity of operation Number of procedures^a^ Blood loss Peritoneal contamination Malignancy status CEPOD status^a^

^a^Graded out of three, remainder graded out of 4

A recent systematic review of patients undergoing emergency abdominal surgery identified 25 risk assessment tools in 20 studies (published between 1993 and 2013) of more than 110,000 patients [16] and reported that APACHE II, ASA, and P-POSSUM scoring systems were the most frequently used scoring systems. The group reported both pre- and post-operative risk assessment at 30 days or in-hospital mortality in a heterogeneous patient group identified from their eligible studies, and so was unable to reliably report on the performance of any tool. ASA grading has been shown to have poor discriminatory performance when used as a comparator for a colorectal surgical risk score in a validation sample of 300 patients aged over 80 requiring emergency colectomy (AUC 0.66) [17]. ASA grade is therefore unlikely to be an appropriate tool in the elderly cohort. The Sickness Assessment is a less well-known score used in the over 65 age group. It includes the parameters: hypotension on arrival, presence of chronic disease and degree of self-caring [18]. Importantly, this includes social components which may reflect markers of frailty which are not included in more general scores.

The P-POSSUM score has been suggested to have predictive utility in vascular patients [19] and has been reported as one of the most frequently utilised in the emergency laparotomy setting [16]. But how well does it predict inpatient mortality in those aged over 70 years? The primary study objective was to determine whether or not risk stratification, based on the P-POSSUM score, reliably predicted inpatient mortality in elderly patients when used in the pre-operative setting.

Good care was reported in only 36 % of elderly patients who died within 30 days of surgery [5]. It is possible that the time taken for key management decisions (surgical review, decision to operate, consultant surgical review, antibiotic prescription and laparotomy) maybe unacceptably delayed in these patients. The secondary objective was therefore to establish whether those who died at 30 days had a delay in any key management steps compared to survivors.

## Methods

This cohort study used NELA data collected from a large tertiary university hospital, with approval for NELA data analysis prospectively granted through the hospital NELA lead as a service evaluation. Consecutive hospital admissions were recorded, and patients were eligible if they were aged 70 or over when admitted as an emergency between 02 January 2014 and 25 August 2015, then underwent an emergency laparotomy. Patients were followed up until discharge or in-hospital death. Data collection was overseen by a hospital lead investigator; surgeons and anaesthetists involved with included cases were responsible for data entry using the NELA audit data web tool [20].

Outcomes were 30-day inpatient mortality and time to death or discharge. All patients were reviewed by the surgical team and underwent an emergency laparotomy. The P-POSSUM and ASA scores were calculated by the anaesthetist in change of the case, and interventions were performed by the duty surgical, anaesthetic and theatre teams. The pre-operative P-POSSUM scores were used for analyses throughout. The information source was the local NELA database. Data were entered onto this from biochemistry, haematology and histology databases at the time of intervention. A power calculation was performed to ensure an adequate sample size was included (based on a 30-day mortality of 15 % (to account for regional variation in mortality), a standard deviation of 0.5, an *α*-error of 0.05 and *β*-error of 0.80). A minimum of 69 patients were required to test the null hypothesis that risk stratification, based on the P-POSSUM score, does not reliably predict inpatient mortality in elderly patients when used in the pre-operative setting.

Quantitative variables such as P-POSSUM score was grouped as low (<5 %), medium (≥5 %, <10 %) and high (≥10 %) for the purpose of developing contingency tables. Patients who remained as inpatients for more than 60 days were removed from the survived to discharge analysis but included in all others. Statistical tests were performed using IBM SPSS Statistics for Windows, Version 22.0. Armonk, NY: IBM Corp. Data were assessed for normality by distribution; then, parametric or nonparametric (Pearson Chi-Square, and Mann–Whitney U) tests and correlations (Pearson’s r and Spearman Rank) were used as appropriate. Correlations between not only the pre-operative P-POSSUM score but also, patient age, white cell count (WCC), creatinine, urea, haemoglobin, heart rate, systolic blood pressure, and lactate with the number of days to death were performed, to identify whether any other variable strongly correlated with time to death, and therefore might be predictive in its own right. Pre-operative P-POSSUM score was assessed by 30-day mortality and survival to discharge outcomes. ROC and AUC were calculated by assigning whether cases were dead or alive at 30 days and plotting against the value for the P-POSSUM and ASA score in the form of sensitivity and 1-specificity. Multivariate regression was not performed on P-POSSUM covariates.

Time from admission to surgical review, decision to operate, consultant surgical review, antibiotic prescription, laparotomy and discharge or death were recorded, and *t* tests were performed to compare the 30-day survival and non-survival groups at each of these points. Other time-critical management steps which did not apply to all patients in the cohort (such as intensive care admission time) were not included. Graphs were constructed using GraphPad Prism version 5 for Windows, GraphPad Software, La Jolla California, the USA. The Strengthening the Reporting of Observational Studies in Epidemiology (STROBE) [21] guidelines were adhered to conduct the study.

## Results

One hundred and ninety-three patients aged 70 or over were admitted and underwent an emergency laparotomy between 02 January 2014 and 25 August 2015. The baseline characteristics and age distribution are shown in Table 2. Of these, 81.9 % (158) survived to discharge, and 87.6 % (169) survived for 30 days. Three patients were inpatients for 60 days.

**Table 2:** Population characteristics

Characteristic	Outcome
Baseline characteristics	
Age	Mean 79.8 (SD 6.2) Median 80
Male sex	47.7 %
P-POSSUM median	27.5 (SD 28.7)
Pre-operative	13.0 (IQR 5.0–40.0)
Post-operative	12.6 (IQR 4.9–46.3)
Key procedure at laparotomy	
Small bowel resection	23.3 % (45)
Hartmann’s procedure	15.0 % (29)
Other large bowel resection	16.6 % (32)
Adhesiolysis	14.0 % (27)
Other procedure	31.1 % (60)
Contamination	
Peritoneal contamination present	40.1 % (78)
Of which generalised contamination	56.4 % (44)
Underlying pathology	
Histologically proven malignancy	24.9 % (48)
Ischaemia	19.7 % (38)
Not applicable or other	55.4 % (107)
Survival	
Survived to discharge^a^	81.9 % (158)
30-day survival overall	87.6 % (169)

^a^By 60 days

**Fig. 1:**
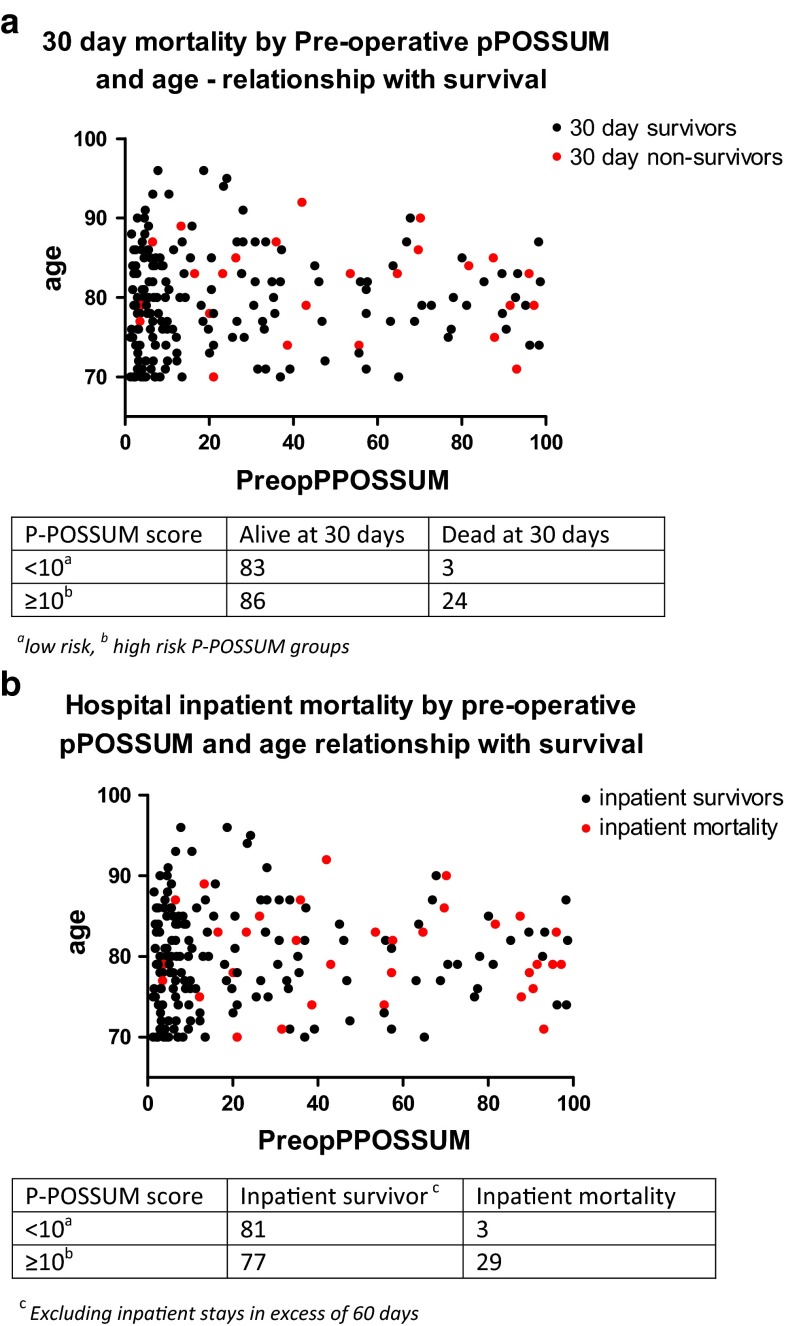
**a** Pre-operative P-POSSUM is associated with 30-day mortality in the over 70 cohort. **b** Pre-operative P-POSSUM is associated with inpatient mortality in the over 70 cohort

**Fig. 2:**
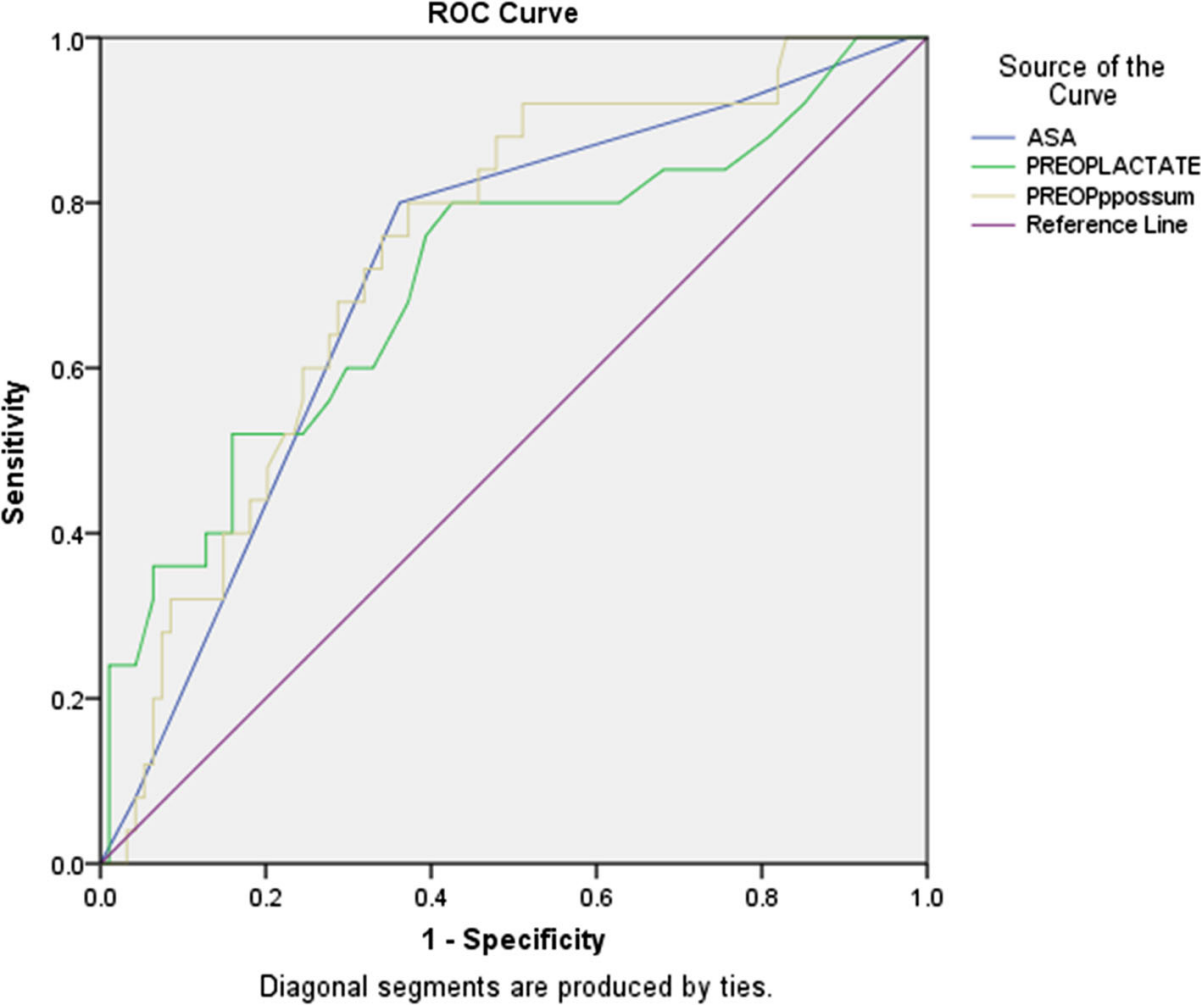
ROC–ASA, P-POSSUM and pre-operative lactate

**Fig. 3:**
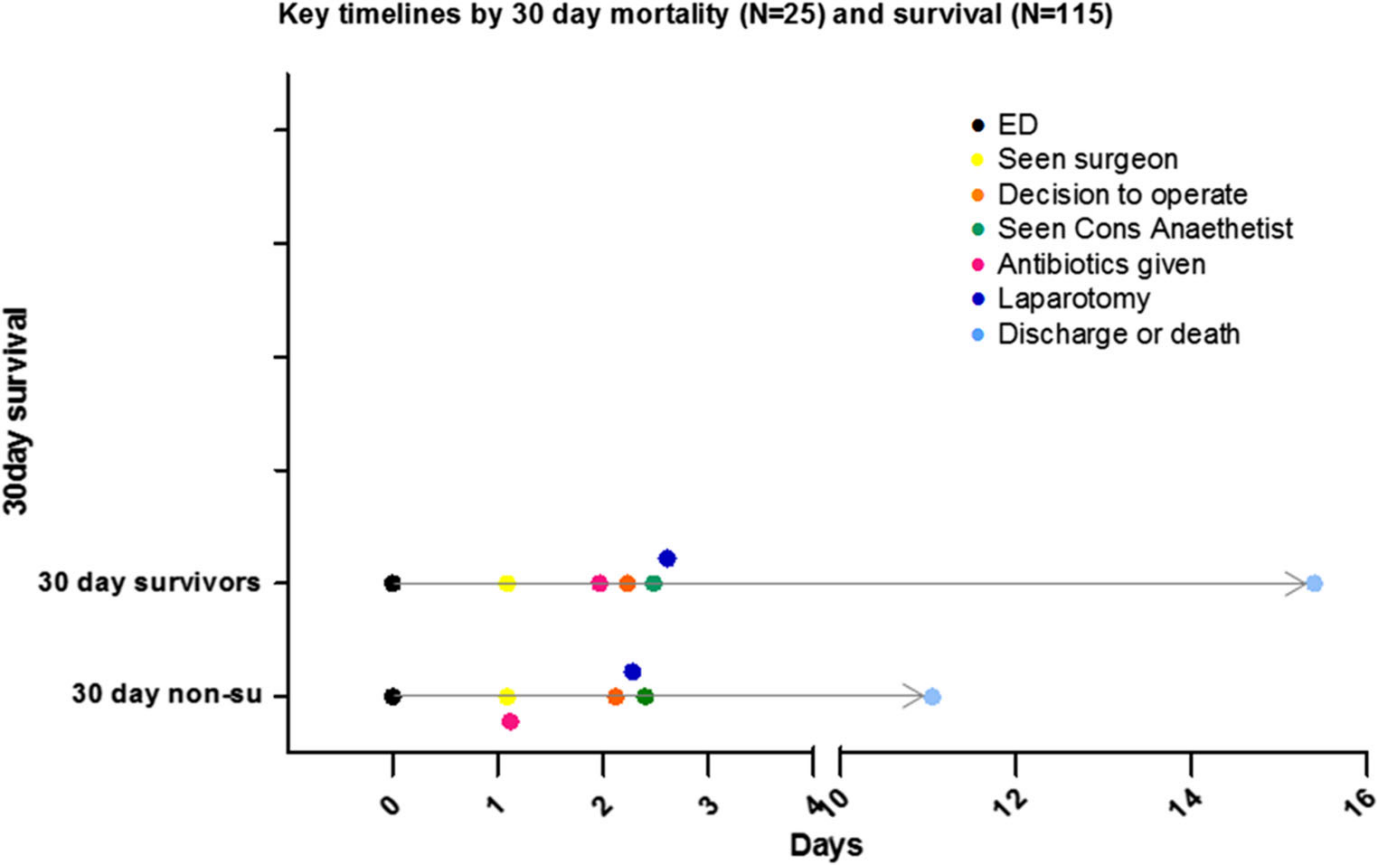
30-day survivors and non-survivors key timelines

Pre-operative P-POSSUM scores exhibited a considerable positive skew; therefore, non-parametric tests were used. When 30-day mortality was compared in low (<5 %), and medium (≥5 %, <10 %), risk versus high (≥10 %) risk P-POSSUM scoring groups, a significant difference was shown (Chi-square pre-operative P-POSSUM *p* = 0.001), and also seen in ungrouped analysis (*p* < 0.001), suggesting that P-POSSUM scores may predict outcome. Figure 1a shows non-survivors to be randomly distributed, whilst half of the survivors fell into the lower (<10 %) risk categories. This may indicate that the tool can discriminate survivors from non-survivors in the lower risk group but performs less well in the higher risk group. Likewise, survival to discharge was significantly different in grouped [Chi-squared (*p* < 0.001)] and ungrouped (Kruskal–Wallis (*p* = 0.006)) testing (Fig. 1b). However, no significant correlation was seen between pre-operative P-POSSUM score and number of days to death (Spearman Coefficient = −0.228, *p* = 0.209). Despite this, pre-operative lactate and haemoglobin did independently correlate with the number of days to death (*p* = 0.003 and 0.022, respectively; Table 3). A receiver–operator curve was generated to assess the predictive capacity of P-POSSUM and ASA scoring (AUC 0.784 and 0.771, respectively, *p* < 0.001), and pre-operative lactate (*N* = 119) (AUC 0.705, *p* = 0.001; Fig. 2). The median lactate in the 30-day survivors and 30-day mortality group was 1.4 (IQR 1.10–2.30) and 2.75 (IQR 1.83–7.35), respectively, and lactate levels significantly differed between those who survived and those who did not at 30 days (*p* = 0.001). When assessing differences in timelines between those who died at 30 days and those who survived, there were no differences in the timing of key decisions (Fig. 3).

**Table 3:** Parameters and their correlation with days to death in those aged over 70

	Correlation (Spearman)	*P* value
Pre-operative P-POSSUM	−0.28	0.21
Age	−0.28	0.13
WCC^a^	0.12	0.52
Creatinine	0.03	0.86
Urea	−0.15	0.41
Haemoglobin	−0.40	0.02 (0.022)
Heart rate^a^	−0.23	0.20
Systolic blood pressure^a^	0.26	0.16
Lactate	−0.57	0.00 (0.003)

^a^Predictors of sepsis [27]

## Discussion

12.44 % of the patients included in this study died within 30 days. This is in line with the national findings of 18 % [3]. The P-POSSUM score is widely adopted in the UK, and its pre-operative use is advocated by the principle investigators of NELA [3]. Its use as a predictive tool through ROC analysis and 30-day survival analysis suggests that this is a moderate discriminator of outcomes (0.7–0.9 AUC), and therefore, it may be used tentatively as a predictor of outcome in those aged over 70.

The AUC was comparable between P-POSSUM, ASA and pre-operative lactate in this study, all being moderate discriminators of outcome. This differs from the findings of others, who have reported ASA scoring to be a poor discriminator in elderly patients undergoing an emergency colectomy (AUC 0.66) [16, 17]. This may reflect underlying population differences. Similarly, studies of emergency colorectal surgery have reported P-POSSUM scoring to be a poor discriminator in colorectal procedures [22, 23]. Intolerance to hypoperfusion in the elderly cohort is highlighted by the correlation between days to death and pre-operative lactate levels in this study. A raised lactate or reduced clearance of lactate consistently predicts a worse outcome in the acute setting and as such may be a useful warning of early mortality in advance of other variables for P-POSSUM scoring (Table 1). However, a normal lactate may not exclude reduced tissue perfusion under certain conditions where there is a lag in the washout of metabolites and may be lower in patients taking drugs such as beta blockers.

In line with good care, NELA investigators recommend that ‘all patients aged over 70 years should undergo an assessment of multimorbidity, frailty and cognition to guide further input from MCOP (Multidisciplinary Teams)’ [3]. MCOP review is not standard practice in the UK acute surgical units, despite frailty being associated with surgical morbidity [24, 25]. The definition of frailty lacks singularity but is broadly synonymous with the concept of ‘reserve’. As such, surgically unwell frail patients require prompt, appropriate decisions and intervention, to optimise survival. Frailty may be considered to be a phenotype including any combination of unintentional weight loss, self-reported exhaustion, grip-strength weakness, slow walking speed and low physical activity, and is independently associated with death [8]. The alternative model of frailty is an accumulation of deficits in the domains of current illness, ability to manage activities of daily living and physical signs over time [26, 27]. Nine frailty tools have been identified for use in surgery globally, but few are used in the emergency setting [26], and a consensus is lacking [28, 25]. Perhaps, a limitation to the current risk assessment tools, such as P-POSSUM, is that frailty is not considered. A suitable frailty screen may provide an additional metric to improve the discriminatory power of the risk assessment tool.

The secondary objective was to establish whether a relationship existed between the timing of key management steps and outcome. The key timelines of those who died at 30 days versus those who did not did not reveal any delay in the former group. It is important to consider that whilst care must be improved for those aged 70 and over, in some, an emergency admission with abdominal ‘catastrophe’ represents an end of life event, and any attempts at rescue may not be in the patient’s best interests. For others, a laparotomy may be their only chance of survival even though the odds of survival are poor. An accurate prediction tool informs such difficult decisions and may encourage shared decision-making between surgical, anaesthetic and critical care teams. If intervention proceeds with high risk, then appropriate planning of post-operative resources and ceiling of care can be considered in advance.

The main study limitation was that mortality data represented inpatient 30-day mortality and did not include patients who were discharged home and subsequently died. The other limitation to full interpretation of results was at lack of frailty scoring. Therefore, frailty for the cohort was unknown and may differ from other regional cohorts. Whilst C-reactive protein (CRP) may have a role for predicting those who are unlikely to have abdominal infections following surgery [29], there is no strong evidence to suggest it predicts mortality following laparotomy and it has not been assessed here. There may be some reporting bias introduced to the NELA data recording in the perioperative phases of surgeon and anaesthetist self-reporting. Individual variability in surgical outcomes may be a source of bias and was addressed as much as possible by the inclusion of consecutive patients over many months and hence normalising the individual reporting and outcome variability. Reporting and selection bias was kept to a minimum as reporting standards have been prospectively established through the audit framework [20]. The outcomes reported have good external validity, due to the nature of data collection and population sampled.

Overall, this study sought to stratify outcomes based on risk. The key finding was that pre-operative P-POSSUM and ASA scoring predicted mortality as moderate discriminators in elderly patients undergoing an emergency laparotomy. Interestingly, pre-operative lactate levels in isolation were likewise able to predict mortality. However, the addition of frailty scoring in conjunction with P-POSSUM and MCOP review in this high-risk group might better identify those with a high risk of mortality after emergency laparotomy and would be a fertile area for further research. Frailty may be a risk factor for both mortality and poor functional recovery after major surgery. Further qualitative studies to assess the impact of emergency laparotomy on the frail patient are needed. To this end, the Fried scoring system [8] (weight loss, weakness, self-reported exhaustion, slowness and low activity) will be validated in our acute general surgery unit.
